# Two Sides of Workplace Interactions: How Appreciation and Social Stressors Shape the Relationship Between Job Insecurity and Well-Being

**DOI:** 10.5964/ejop.v16i3.2023

**Published:** 2020-08-31

**Authors:** Mauricio E. Garrido Vásquez, Patricia Garrido-Vásquez, Kathleen Otto

**Affiliations:** a Department of Psychology, University of Concepción, Concepción, Chile; b Department of Work and Organizational Psychology, Philipps University of Marburg, Marburg, Germany; University of Wollongong, Wollongong, Australia

**Keywords:** job insecurity, appreciation, social stressors, resources, professionals, well-being

## Abstract

Job insecurity has frequently been shown to have a dysfunctional impact on well-being. Based on the conservation of resources (COR) theory, the aim of this study was to investigate how the experience of appreciation at the workplace and the occurrence of social stressors shape the relationship between job insecurity and three indicators of well-being: (a) job satisfaction, (b) (emotional) irritation, and (c) engagement (dedication to the job). In an online study with 117 psychologists, we found that appreciation buffered the relationship between job insecurity and irritation. Social stressors further qualified the moderating effect of appreciation on job satisfaction and dedication, but not fully in the proposed direction. Theoretical implications about the role of more or less social contacts at work (reflected in the experience of appreciation as well as social stressors) when dealing with job insecurity will be discussed.

Today’s professionals face many challenges: Information technology and increasing global competition have brought permanent changes for organizations and individual careers (e.g., Brown, 1995; de Vries & Balazs, 1997; Sverke, Hellgren, & Näswall, 2002), often resulting in uncertainty about the continuity of a (current) job situation. Such uncertainty is known as job insecurity. Research to date has either explored job insecurity across organizations in homogeneous sectors (e.g., Piccoli & De Witte, 2015) or used convenient samples with heterogeneous sectors and professions (e.g., Garrido Vásquez, Truyen, & Otto, 2017; Vander Elst, De Cuyper, Baillien, Niesen, & De Witte, 2016). Here, we address job insecurity perceptions in one specific group of professionals: psychologists. Thereby, we ensure a comparable educational and professional background. Although various specializations exist (e.g., psychotherapy in a hospital or practice, academic research, consultants in companies), prior research has found roles, functions, and purposes of the work of psychologists to be comparable (Bartram & Roe, 2005). Regarding job insecurity, psychologists’ career development is characterized as being *intermittent* or *inconsistent*, because career interruptions or varying professional activities are quite usual (Hoff, Grote, & Wahl, 2002).

Research on job insecurity has provided comprehensive evidence about its negative consequences (e.g. De Witte, 2005), but also searched for variables that could increase or decrease the effects of job insecurity. Intervening variables that have been proposed so far are demographic variables (e.g., age or gender; Cheng & Chan, 2008; De Witte, 1999; Näswall, Sverke, & Hellgren, 2005), personality indicators (e.g., locus of control or flexibility; König, Debus, Häusler, Lendenmann, & Kleinmann, 2010; Otto, Hoffmann-Biencourt, & Mohr, 2011), and type of formal contract or organizational change (Ashford, Lee, & Bobko, 1989; Keim, Landis, Pierce, & Earnest, 2014). Moreover, on a theoretical level Sverke and Hellgren (2002) proposed in their integrated model of job insecurity that social support buffers the relationship between job insecurity and its negative consequences. Empirical evidence has indicated that social factors at work, in particular social support, might work as a moderator: For instance, Lim (1996) found that non-work based social support moderated the relationship between job insecurity and job dissatisfaction. However, there is still very little evidence on the role of social factors besides social support for dealing with job insecurity (Lim, 1996; Sverke et al., 2002).

Daily social contacts can bring both positive and negative experiences. The former may help deal with challenging working conditions such as the perception of job insecurity (Lim, 1996), whereas the latter could probably further aggravate these bad conditions. To the best of our knowledge, research so far has explored the role of the social surrounding for job insecurity either from the perspective of a potential buffer (i.e., social support; Lim, 1996) or stressor (e.g., Garrido Vásquez, Kälin, Otto, Sadlowski, & Kottwitz, 2019). Our study is the first to simultaneously take into account the role of both sides of social interactions, thereby generating a more complex picture of the effects of the social surrounding on coping with job insecurity.

A first aim of the present study was to investigate the effects of *appreciation* (for one’s job) as a hitherto neglected social resource. Because providing appreciation can be seen as a core aspect of the psychological profession itself, it is reasonable to assume that perceiving appreciation also works as a resource for psychologists themselves. Hence, we investigated whether appreciation could buffer the negative effects of job insecurity for psychologists.

*Social stressors* are located on the negative side of the social surrounding at work. The literature on social stressors, defined as “social animosities, conflict with coworkers and supervisors, unfair behavior, and a negative climate” (Dormann & Zapf, 2002, p. 35), has shown that conflicts with coworkers, supervisors, or customers have negative consequences on workers’ health (e.g., Dormann & Zapf, 2002; Otto & Mamatoglu, 2015). Thus, a second aim of this study was to investigate whether the proposed buffering effect of appreciation on job insecurity remains robust when psychologists experience social stressors at work.

## Conservation of Resources Theory, Job Insecurity, and Well-Being

According to the conservation of resources (COR) theory (e.g., Hobfoll, 1989, 2001; Hobfoll & Shirom, 2001), individuals strive to obtain, retain, foster, and protect those things in life they centrally value. These valuable things are called resources. Within this framework, stress appears as a reaction to an environment in which individuals (a) are threatened by a loss of resources, (b) actually lost resources or (c) did not obtain the resource they were striving for. Resources are either valued in their own right (so-called primary resources such as positive social relationships or self-esteem), or they are necessary in order to achieve or protect valued resources (secondary resources). One’s job could be seen as an important secondary resource (c.f., Semmer, Grebner, & Elfering, 2010) that has to be protected: Beyond earning money, it provides significant positive psychosocial functions, such as maintaining and advancing our skills, giving us structure, facilitating social contacts, and appreciation. Moreover, it often is an important part of our identity (Jahoda, 1983).

Resources are used to prevent resource loss. Gaining resources is particularly important after the failure of an investment or as compensation in the context of losses (Hobfoll, 1989, 2001). Due to resource losses, loss spirals can develop which aggravate the situation. More importantly, the model discusses the consequences of resource losses in general; thus an event at work can be interpreted as stressful because it represents a threat to the continuity of one’s resources. Within the scope of job insecurity, there might be resources, the loss of which increases the perception of the likelihood of losing one’s job. Applying the COR theory to our study, we argue that one’s job is an important resource that has to be protected, and job insecurity has negative consequences on employees’ well-being because it constitutes a threat to the continuity of this important resource.

Lazarus and Folkman (1984) proposed that the effects of anticipating a negative event could be equal or even worse than the effects of the event itself. In other words, the effects of job insecurity could be comparable or even worse than those of actual unemployment (Otto & Dalbert, 2013). Several studies have shown that job insecurity has negative consequences on workers’ health (Burgard, Brand, & House, 2009; Çetin & Turan, 2013; Dekker & Schaufeli, 1995; Hellgren & Sverke, 2003; Hellgren et al., 1999): It impairs well-being (Hellgren, Sverke, & Isaksson, 1999) and leads to mental health complaints (Hellgren & Sverke, 2003), as well as irritation (De Cuyper & De Witte, 2005). In the present study, as indicators of well-being we included job satisfaction, emotional irritation, and engagement, assuming that job insecurity is negatively related to job satisfaction (Sverke et al., 2002) and positively related to irritation (Büssing, 1999; De Cuyper & De Witte, 2005; Otto et al., 2011). The positive psychology literature has furthermore classified engagement as an important indicator of well-being (Bakker, Schaufeli, Leiter, & Taris, 2008; Ouweneel, Le Blanc, & Schaufeli, 2013).

Engagement is understood as the antithesis of burnout and is defined as a positive, fulfilling, work-related state of mind that is characterized by vigor, dedication, and absorption (Schaufeli, Bakker, & Salanova, 2006). In this article we focused on one dimension of engagement: dedication. Schaufeli and Salanova (2008) argued that dedication refers to “being strongly involved in one’s work, and experiencing a sense of significance, enthusiasm, inspiration, pride, and challenge” (p. 381-382). Moreover, dedication is described as an emotional component of engagement. When thinking about the job description of psychologists, “helping people” might be one of the first associations which comes to mind. Dedication therefore seems to be part of the psychological profession. Job insecurity is negatively related to engagement (Bosman, Rothmann, & Buitendach, 2005; De Cuyper & De Witte, 2005; Stander & Rothmann, 2010). In a longitudinal study Mauno, Kinnunen, and Ruokolainen (2007) found that job insecurity decreased dedication at work after controlling for the baseline level of dedication. Accordingly, we expected that job insecurity would not only have an impact on job satisfaction and emotional irritation, but also on dedication to the job.

## Positive Social Interactions: Appreciation as a Job Resource

Psychologists work in different fields. Bartram and Roe (2005) found that these different fields, however, have something in common, namely that psychologists work with people, either with individuals (e.g., conducting a therapy), with loose groups (e.g., teaching classes to students), or with fixed teams (e.g., doing trainings in organizations). Thus, social interactions are part of the job of psychologists (Otto et al., 2019). Sobiraj, Schladitz, and Otto (2016) suggested that psychologists put a high value on receiving appreciation, which could be considered one of the most striking aspects of human nature. Indeed, some evidence indicates that appreciation can be classified as the most essential of all accessible rewards for workers (van Vegchel, De Jonge, Bakker, & Schaufeli, 2002). Essentially, COR theory has proposed that gaining new resources becomes increasingly important when other resources (here: a secure job) have been lost in stress situations (Hobfoll, 2002). Hence, receiving appreciation might counteract the imminent resource loss. Appreciation is considered an important resource at work which shows, for example, positive correlations with job satisfaction (Jacobshagen & Semmer, 2009; Semmer, Jacobshagen, Meier, & Elfering, 2007; Stocker, Jacobshagen, Semmer, & Annen, 2010). A longitudinal study with young workers found that participants who reported high appreciation were more satisfied, and that the most important source of appreciation was their supervisors, followed by clients (Semmer, Tschan, Elfering, Kälin, & Grebner, 2005).

The literature has reported that job resources buffer the impact of job demands (Bakker, Demerouti, & Euwema, 2005; Bakker et al., 2007; Stocker et al., 2019). Stocker et al. (2019) found that appreciation, as a job resource, buffered the negative impact of work interruptions on employees´ well-being. In a sample of teachers, Bakker, Hakanen, Demerouti, and Xanthopoulou (2007) found that appreciation was positively related to work engagement, and that appreciation moderated the relationship between pupil misbehavior and work engagement. These studies highlight the potential of appreciation for reducing the impact of job demands at work. In our study, if through job insecurity a resource loss is at stake, we argue that appreciation might shield against its negative consequences on well-being (job satisfaction and emotional irritation) and also play a role for dedication to one’s job. Therefore, we expected appreciation, which is an important job resource for psychologists (Sobiraj et al., 2016), to moderate the relationship between job insecurity and its consequences.

H1a: Appreciation weakens the negative relationship between psychologists’ job insecurity and their job satisfaction.

H1b: Appreciation weakens the positive relationship between psychologists’ job insecurity and their (emotional) irritation.

H1c: Appreciation weakens the negative relationship between psychologists’ job insecurity and their dedication towards the job.

## Negative Social Interactions: Social Stressors and (Further) Resource Loss

As outlined before, the psychological profession involves contact with other people, either supervisors, coworkers, or, depending on the field (i.e., therapist or consultant), with clients or patients. Thus, social interactions are part of everyday life for many psychologists (Otto et al., 2019). When these social interactions are affected by conflicts or a negative climate, they might be perceived as social stressors. Social stressors are a common situation in the work environment (e. g., Eurofound, 2017; Schwartz & Stone, 1993). Findings have indicated that the most common daily problems reported by participants were conflicts with supervisors, colleagues, and/or customers (Schwartz & Stone, 1993).

Social stressors have negative effects not only on workers` health (Dormann & Zapf, 2002; Otto & Mamatoglu, 2015), but also on attitudinal and behavioral outcomes (Bruk-Lee & Spector, 2006; Grebner et al., 2003; Hershcovis & Barling, 2010; Kessler, Spector, Chang, & Parr, 2008). According to Hobfoll (2011; Hobfoll, Halbesleben, Neveu, & Westman, 2018) an important principle of the COR theory is the primacy of resource loss. It means that resource loss is more salient than resource gain. If we consider the detrimental effects of job insecurity and social stressors, it would be expected that both stressors together have a negative impact on individuals, because they might threaten the continuity of an important resource, namely a secure job. A second principle of the theory is resource investment (Hobfoll, 2011; Hobfoll et al., 2018). This principle emphasizes the importance of resource investment in order to protect against resource loss. Both Hobfoll (2011) and Hobfoll et al. (2018) have argued that people with greater resources are less vulnerable to resource loss and more capable to gain resources than people with fewer resources, but this requires that they either build new resources or have resources at their disposal.

Taking into account the negative effects of social stressors, the positive effects of appreciation, and the principles mentioned above, we expected that social stressors would aggravate the negative impact of job insecurity (job insecurity and social stressors as two work stressors, causing additional resource loss) on outcomes only when psychologists perceive low appreciation, but not when they perceive high appreciation at work.

H2a: The buffering effect of high appreciation on the relationship between job insecurity and job satisfaction remains robust when social stressors occur.

H2b: The buffering effect of high appreciation on the relationship between job insecurity and (emotional) irritation remains robust when social stressors occur.

H2c: The buffering effect of high appreciation on the relationship between job insecurity and dedication remains robust when social stressors occur.

## Method

### Procedure and Participants

We conducted a study with psychology graduates from a German university, who were requested via e-mail to answer an online questionnaire. The link to the online questionnaire was sent to all individuals who had left their email address when they graduated from university. Overall 133 psychologists answered the questionnaire. They had finished their studies between 2001 and 2006. Considering that, on average, 60 psychologists graduated each year from this university, the return rate is around 35% or higher, because not all psychology graduates still use their old student e-mail accounts. From the 133 graduates who answered the questionnaire, 16 (12%) reported that they were currently unemployed, and therefore they were excluded from further analysis. Thus, our sample size comprised 117 psychology graduates, whose ages ranged from 24 to 49 years (*M* = 31.21, *SD* = 4.88). Overall, 82% of the participants were female. Most of the participants worked in the field of clinical psychology (42.7%), followed by research (17.9%), work and organizational psychology (12%), educational psychology (6%), criminal psychology (3.4%), and 17.9% were working in other fields in- and outside the psychological profession.

### Measures

#### Job Insecurity

We assessed job insecurity with a single item taken from the Job Descriptive Questionnaire by Neuberger and Allerbeck (1978; "The risk of losing my job is high"). The item was answered on a 6-point Likert-scale ranging from 1 (*strongly disagree*) to 6 (*strongly agree*). This item measures the cognitive dimension of job insecurity, as opposed to its affective dimension, which assesses worries about the future of one’s job (Borg & Elizur, 1992). It has to be noted that Garrido Vásquez et al. (2019) provided evidence of a substantial correlation between a single-item job insecurity measure and a well-established multiple-item measure.

#### Appreciation

We assessed appreciation with four items based on the Effort-Reward Imbalance (ERI) questionnaire (Siegrist et al., 2004); e.g., “I receive from my colleagues the appreciation that I deserve”, “I receive from my supervisors the appreciation that I deserve”. The items require a response on a 6-point scale that ranges from 1 (*strongly disagree*) to 6 (*strongly agree*). Cronbach's alpha of .66 indicated quite low internal consistency. It has to be taken into account, however, that various sources of appreciation (i.e., coworkers, supervisors, clients/customers, family/friends) were explored, and it seems to be questionable that all sources equally provide appreciation. Moreover, since the measure is quite short, also inter-item correlations should be considered (Streiner, 2003). As a rule of thumb, Clark and Watson (1995) argued that mean inter-item correlations with values of .40 to .50 should be yielded for measures depicting very narrow and of .15 to .20 for measures depicting broad characteristics. With a mean inter-item correlation of .33 our value falls in between these boarders, indicating that the measure is reliable.

#### Social Stressors

Social stressors were explored with eight items of the respective scale developed by Frese and Zapf (1987), which measures conflicts with supervisors and colleagues: e.g., "With some colleagues there is often conflict". The items were answered on a 4-point Likert-scale ranging from 1 (*disagree*) to 4 (*agree*); Cronbach's alpha of .76 indicated satisfactory internal consistency.

#### Job Satisfaction

We measured job satisfaction with the seven global items taken from the Job Descriptive Questionnaire by Neuberger and Allerbeck (1978): e.g., "All in all, I am satisfied with my co-workers", "All in all, I am satisfied with my supervisor", “All in all, I am satisfied with my pay”. The items were answered on a 6-point Likert-scale that ranged from 1 (*strongly disagree)* to 6 (*strongly agree*). Cronbach's alpha of .78 indicated a satisfactory internal consistency.

#### Emotional Irritation

We assessed emotional irritation with a five-item subscale from the Irritation scale developed by Mohr, Rigotti, and Müller (2005), e.g., "From time to time I feel like a bundle of nerves", “I get irritated easily, although I don’t want this to happen”. These items require a response on a 7-point scale that ranges from 1 (*does not apply at all*) to 7 (*applies nearly completely*). Cronbach's alpha of .90 indicated a high internal consistency.

#### Dedication

We measured dedication to one’s job as an indicator of engagement with the respective three items taken from the short form of the Utrecht Work Engagement Scale by Schaufeli and Bakker (2003), e.g., "I am enthusiastic about my job". The items were answered on a 6-point Likert-scale that ranged from 1 (*strongly disagree*) to 6 (*strongly agree*). Cronbach's alpha of .88 indicated a good internal consistency.

### Statistical Analysis

To analyze our assumption of a moderated moderation by appreciation and social stressors on the relationship between job insecurity and job satisfaction, emotional irritation, and dedication, we tested our hypotheses using regression analyses. We centered the predictor and the two proposed moderators before the analyses were conducted (Aiken, West, & Reno, 1991) and tested our hypotheses using the SPSS (Version 24) macro “Process” by Hayes (2013). We included all our variables in a whole model in order to calculate the three-way interactions. The three-way interaction included the interaction between our independent variable and the moderators (job insecurity x appreciation x social stressors). This analysis includes a first interaction between the independent variable and the first proposed moderator (job insecurity x appreciation). A second interaction was tested between the independent variable and the second proposed moderator (job insecurity x social stressors). The third interaction included the two moderators (appreciation x social stressors). Finally, we assessed the three-way interaction with the independent variable and the two moderators (job insecurity x appreciation x social stressors) in order to investigate the intervening effect of appreciation depending on social stressors. According to Baron and Kenny (1986), the interaction must be significant in order to support the moderation hypothesis. If the independent variable or the moderator is not related to the outcome, this result is not relevant for testing the hypothesis. Moreover, we conducted a simple slopes analysis (± 1 *SD* from the average) using Process when the interaction terms were significant (Hayes, 2013).

## Results

Table 1 shows the means, standard deviations, and correlations among the study variables. Both appreciation and social stressors were significantly correlated with job satisfaction, emotional irritation, and dedication, but not with job insecurity. Surprisingly, job insecurity did not show any significant correlations with the dependent variables on a bivariate level.

**Table 1:** Means, Standard Deviations, and Zero-Order Correlations of the Study Variables

Variable	*M*	*SD*	1	2	3	4	5	6
1. Job insecurity	2.54	1.45	-					
2. Appreciation	4.53	0.76	-.02	-				
3. Social stressors	1.58	0.47	.05	-.35**	-			
4. Job satisfaction	4.59	0.81	-.04	.59**	-.41**	-		
5. Emotional irritation	2.72	1.25	.05	-.24*	.38**	-.25**	-	
6. Dedication	4.63	1.00	-.04	.49**	-.19	.57**	-.11	-

*p < .05. **p < .01.

### Regression Analyses

The results of the regression analyses are displayed in Table 2. We examined the moderated effects of appreciation, as well as social stressors, on the relationship between job insecurity and job satisfaction, emotional irritation and dedication.

**Table 2:** Predicting Job Satisfaction, Emotional Irritation and Dedication by Job Insecurity, Appreciation and Social Stressors

Variable	Job satisfaction			Emotional irritation			Dedication		
	*B*	*SE*_B_	*t*	*B*	*SE*_B_	*t*	*B*	*SE*_B_	*t*
Constant	4.59***			2.57***			4.67***		
Job insecurity	-0.07^†^	.05	-1.34	0.02	.09	0.22	-0.13*	.07	-1.91
Appreciation	0.54***	.10	5.58	-0.23^†^	.17	-1.37	0.67***	.13	5.01
Job insecurity x Appreciation	0.06^†^	.04	1.57	-0.19**	.09	-2.17	-0.09*	.06	-1.68
Social stressors	-0.40**	.14	-2.78	0.93***	.23	4.06	0.05	.19	0.27
Job insecurity x Social stressors	0.07	.10	0.46	-0.01	.20	-0.07	-0.13	.16	-0.80
Appreciation x Social stressors	-0.23	.31	-0.73	0.54	.52	1.03	0.22	.36	0.62
Job insecurity x Appreciation x Social stressors	-0.34*	.18	-1.90	0.22	.42	0.53	-0.55*	.30	-1.80

^†^*p* < .10. *p < .05. **p < .01. ***p < .001.

**Figure 1:**
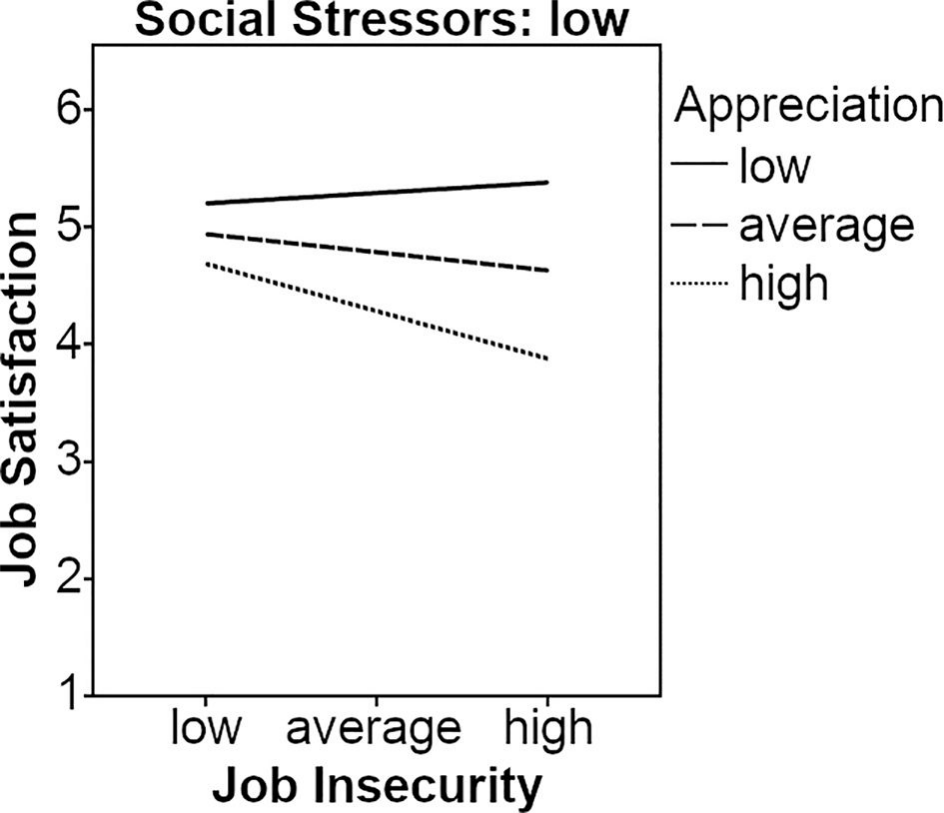
Appreciation and low social stressors as moderators on the relationship between job insecurity and job satisfaction.

**Figure 2:**
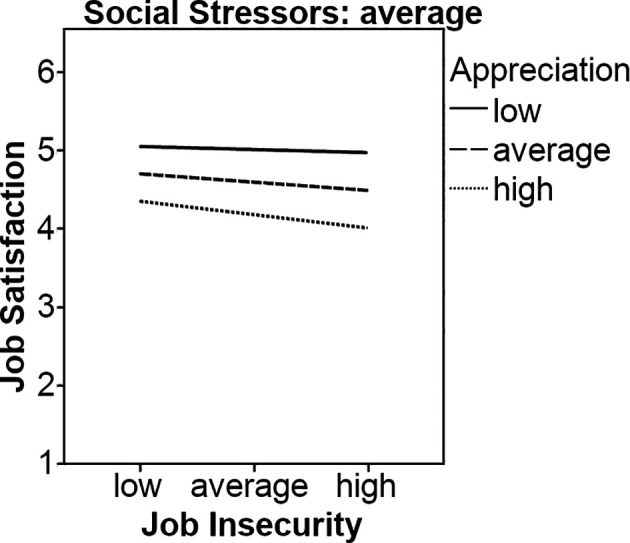
Appreciation and (average) social stressors as moderators on the relationship between job insecurity and job satisfaction.

Job insecurity was negatively related to job satisfaction at trend level (*b* = -0.07, *p* < .10). Appreciation was positively (*b* = 0.54, *p* < .001), and social stressors were negatively related to job satisfaction (*b* = -0.40, *p* < .01). The interaction between job insecurity and appreciation for predicting job satisfaction was marginally significant (*b* = 0.06, *p* < .10). Simple slopes analyses showed that neither the line representing high appreciation (*b* = -0.002, *ns*) nor the line representing low appreciation (*b* = -0.05, *ns*) were significant, therefore Hypothesis 1a had to be rejected. The three-way interaction was significant and negatively related to job satisfaction (*b* = -0.34, *p* < .05). Nevertheless, our results indicated that the line representing low appreciation was significant (*b* = -0.27, *p* < .05), and the line representing average appreciation was marginally significant (*b* = -0.11, *p* < .10) when social stressors were low. When social stressors were average, the line representing low appreciation was significant (*b* = -0.11, *p* < .05), and the line representing average appreciation was marginally significant (*b* = -0.07, *p* < .10). There were no significant results when social stressors were high, providing partial support for Hypothesis 2a only, see Figure 1 and Figure 2.

**Figure 3:**
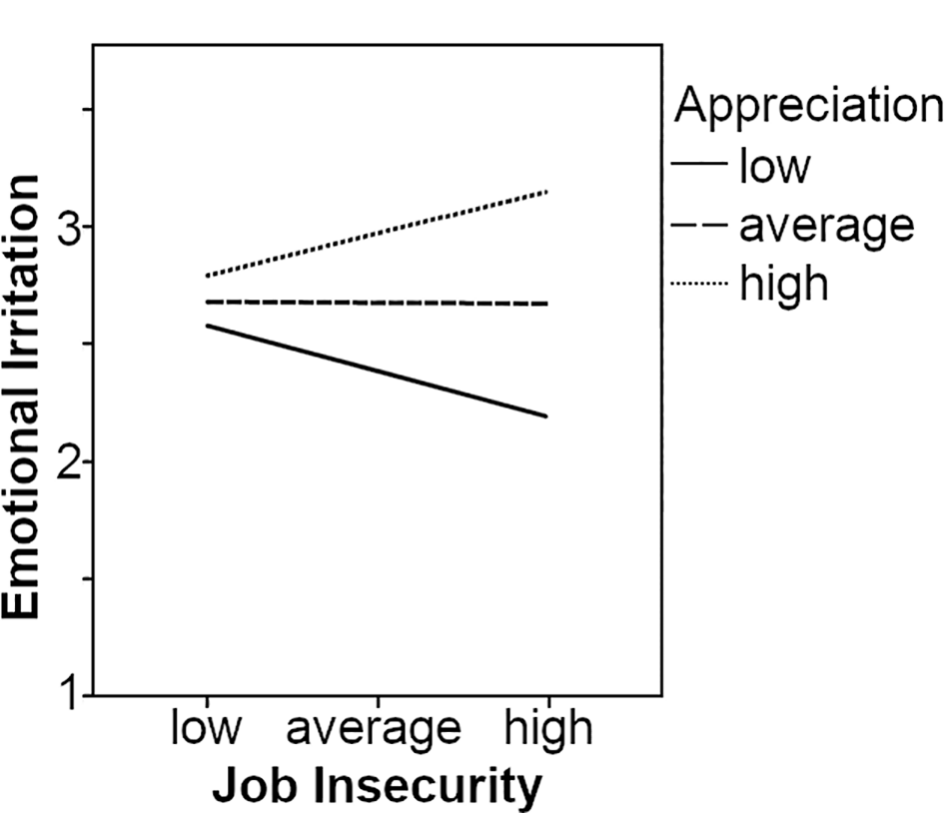
Appreciation as a moderator on the relationship between job insecurity and emotional irritation.

Next, we examined the effects of appreciation and social stressors on the relationship between job insecurity and emotional irritation. Job insecurity was unrelated to irritation (*b* = 0.02, *ns*). Appreciation (*b* = -0.23, *p* < .10) was marginally negatively related, and social stressors were positively related to irritation (*b* = 0.93, *p* < .001). The interaction between job insecurity and appreciation was significant, and it was negatively related to emotional irritation (*b* = -0.19, *p* < .05). Simple slopes analyses, illustrated in Figure 3, showed that both the line representing high appreciation (*b* = -0.13, *p* < .10) and the line representing low appreciation (*b* = 0.12, *p* < .10) were marginally significant. For high appreciation, as proposed, a negative relation was found. Thus, our results provided some support for Hypothesis 1b. The three-way interaction was not significant (*b* = 0.22, *ns*), and therefore Hypothesis 2b had to be rejected.

**Figure 4:**
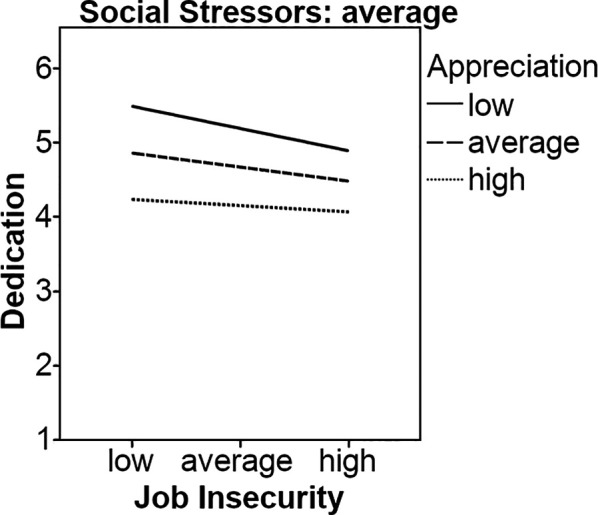
Appreciation and average social stressors as moderators on the relationship between job insecurity and dedication.

**Figure 5:**
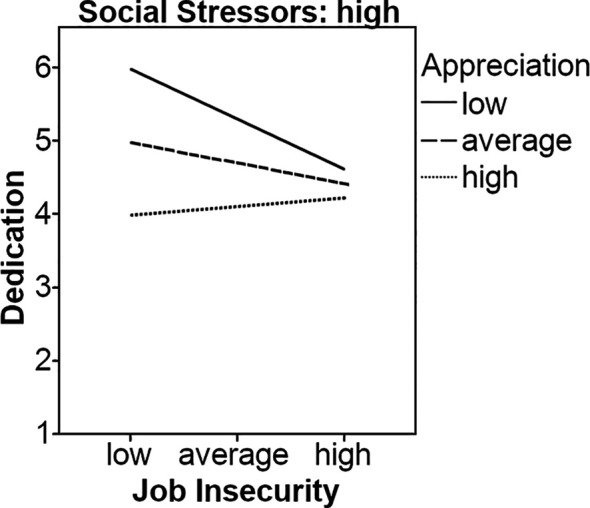
Appreciation and high social stressors as moderators on the relationship between job insecurity and dedication.

Finally, we also analyzed the effects of appreciation and social stressors on the relationship between job insecurity and dedication towards one’s job. Our results indicated that job insecurity (*b* = -0.13, *p* < .05) and appreciation (*b* = 0.67, *p* < .001) were negatively and positively related to dedication, respectively, while social stressors were not (*b* = 0.05, *ns*). The interaction between job insecurity and appreciation on predicting dedication was significant (*b* = -0.09, *p* < .05). However, simple slopes analyses showed that neither the line representing high appreciation (*b* = -0.10, *ns*) nor the line representing low appreciation (*b* = 0.01, *ns*) was significant, therefore Hypothesis 1c had to be rejected. The three-way interaction was significant and negatively related to dedication (*b* = -0.55, *p* < .05). Nevertheless, our results showed that the lines representing average appreciation (*b* = -0.12, *p* < .05) and high appreciation (*b* = -0.20, *p* < .01) were significant when social stressors were reported to be on average. When social stressors were high, the lines representing average (*b* = -0.19, *p* < .05) and high appreciation (*b* = -0.46, *p* < .05) were also significant. However, there were no significant results when social stressors were low, providing at least some support for Hypothesis 2c, see Figure 4 and Figure 5.

## Discussion

Building on the COR theory (e.g., Hobfoll, 1989, 2001; Hobfoll & Shirom, 2001), the aim of the present study was to investigate the buffering role of appreciation as a potential resource in the relationship between job insecurity and three indicators of well-being: job satisfaction, emotional irritation, and dedication. Because in social professions positive social interactions might co-occur with negative social interactions, a second aim of our study was to explore whether the positive effects of appreciation remain robust when psychologists experience social stressors at work. Our results confirmed appreciation as a significant moderator in the relationship between job insecurity and its outcomes (counteracting the resource loss brought by job insecurity), while social stressors further impacted on the moderated effects of appreciation—though not always in the way we expected.

### The Interplay of Appreciation and Social Stressors for Job Satisfaction and Dedication

In this study we simultaneously investigated the role of appreciation and social stressors on the relationship between job insecurity and well-being. In our first set of hypotheses we proposed that appreciation would buffer the negative relationship between job insecurity and job satisfaction and between job insecurity and dedication. However, this simple buffering effect of appreciation was not confirmed. According to the COR theory (e.g., Hobfoll, 1989, 2001; Hobfoll & Shirom, 2001), social relations are a resource, which can provide or facilitate the preservation of valued resources. These effects might be positive when they are provided/facilitated for situational needs, but negative when they are not (Hobfoll, 1989). As already mentioned, resources are important because they can protect other valued resources or lead to the gain of new ones. Studies based on the COR theory (e.g., Hobfoll, 1989, 2001; Hobfoll & Shirom, 2001) have suggested that resources might buffer negative effects on well-being (e.g. sources of social support; Halbesleben, 2006).

In the present study, the role of appreciation turned out to be relevant for both job satisfaction and dedication when simultaneously considered with social stressors. Based on COR theory, we originally assumed that high appreciation (resource gain) could buffer the negative effects of job insecurity (resource loss) on job satisfaction and dedication even when psychologists have conflicts at work (social stressors, i.e. resource loss). Our findings revealed that the three-way interaction for explaining job satisfaction was significant, but only the slope representing low and average appreciation was substantial when the levels of social stressors were also low or average. Those psychologists who received low appreciation by their clients or patients, colleagues or supervisors, and at the same time were confronted with low social stressors were less satisfied when they were experiencing high job insecurity. As situations of low appreciation and low social stressors might reflect overall low social contacts at work, this could be an additional stressor for people who chose to work in a social profession.

Hobfoll (1989, 2001) argued that not only the actual loss of resources is sufficient for producing stress, but also a lack of them. Taking into account the COR theory (e.g., Hobfoll, 1989, 2001; Hobfoll & Shirom, 2001), lack of social contacts (low appreciation, low social stressors) might reduce job satisfaction, because people might feel that they cannot do anything against an eventual further loss of resources, which job insecurity represents. This could lead to a “loss spiral”. Loss spirals follow initial loss, with each loss leading to a further depletion of resources for confronting the next threat or loss (Hobfoll & Shirom, 2001). Notably, not only when resources are available could they have positive effects, but also the lack of resources (here: being isolated with few social contacts) could have opposite effects by increasing the negative consequences of job insecurity as an additional stressor.

Regarding dedication, we had proposed that high appreciation buffers the negative effects of job insecurity on dedication, even when individuals are experiencing social stressors at work. Our results revealed that the proposed three-way interaction was significant, but the simple slope analyses showed that the lines representing average and high appreciation were significant only when the level of social stressors was average or high. These results indicate that appreciation cannot buffer the effects of job insecurity when social stressors are high. We found that the effect of the two-way interaction (job insecurity x appreciation) on dedication was weaker than the effect of job insecurity on the same outcome, suggesting a buffer effect of appreciation. However, when social stressors emerged, job insecurity exerted a stronger negative effect on psychologists’ dedication, even when they felt highly appreciated for their work by their social surrounding. According to COR theory (e.g., Hobfoll, 1989, 2001; Hobfoll & Shirom, 2001), the common occurrence of high job insecurity and high social stressors are linked in a kind of loss spiral. In case of job insecurity, a loss spiral would develop because the affected person, who feels strained because of the perception to lose the job, lacks the resources to offset this loss. Thus, the confrontation with conflicts at work (social stressors) further aggravates this situation. In a recent study Garrido Vásquez et al. (2019) found, for example, that social stressors lead to higher job insecurity perceptions. As a consequence, stress reactions could be evoked, reducing dedication towards one’s job because these stressors overwhelm personal limits and abilities, and people might feel that they are unable to protect themselves from a further loss of resources. In this case, the presence of high appreciation (resource) seems to be insufficient for psychologists to counteract the negative effects of high job insecurity and high social stressors on dedication.

Overall, our results for the three-way interactions for the outcomes job satisfaction and dedication, showed some unexpected and equivocal results. Job insecurity predicted job satisfaction only when appreciation and social stressors were low or average, and it predicted dedication only when appreciation and social stressors were average or high. Thus, it seems that the less social contact (reflected here via appreciation and social stressors) psychologists have, the stronger is the negative association of job insecurity and job satisfaction. On the other hand, for the relationship between job insecurity and dedication, the more social contact psychologists have, the worse appears to be the perception of job insecurity for this relationship. It seems that for psychologists, whose jobs are classified as working in a social sector with people (Bartram & Roe, 2005), the experience of job insecurity strongly affects their job satisfaction when the frequency of social contacts – i.e., experiencing appreciation but also conflicts – decreases. This might indicate that the lack of social contacts in this social profession causes an additional stressor besides job insecurity, and that both stressors (lack of social contact, job insecurity) simultaneously (loss spiral) have the strongest consequences for job satisfaction. On the other hand, being confronted with conflicts and receiving appreciation at the same time – both being indicative of many social contacts – job insecurity negatively affects a psychologist’s engagement (i.e., dedication). Future research should consider these results in order to better understand the mechanism behind these results, for instance differentiating between professional groups in order to investigate whether these results could be generalized to different professions, or if they could only be expected for employees working in social professions.

Finally, another point for future research is the role of frequency or intensity of social contacts. Our study revealed that the frequency of social contacts of psychologists (operationalized via appreciation and social stressors) affected the level of job satisfaction and dedication they reported. Therefore, future research should investigate whether the frequency or intensity of social contacts is essential in the relationship between job insecurity and its consequences. This would go beyond appreciation and social stressors at work.

### The Interplay of Appreciation and Social Stressors for Emotional Irritation

In this study we explored appreciation as a moderator in the relationship between job insecurity and emotional irritation. We proposed that when individuals receive appreciation at work (resource gain), it could decrease the negative consequences of job insecurity (resource loss). Our results indicated that when individuals receive high appreciation, the negative consequences of job insecurity could be decreased. This result shows that job resources (appreciation) could buffer the effects of job insecurity on irritation, providing support for the idea that resources are necessary in order to achieve or protect valued secondary resources, such as one’s job.

Above all, we aimed to investigate whether the buffering effects of high appreciation on emotional irritation caused by job insecurity remain robust when negative social interactions, i.e., social stressors occur. Although we found that appreciation moderated the relationship between job insecurity and irritation when we included social stressors in the model, job insecurity no longer predicted irritation. In our study, social stressors refer to conflicts with supervisors and colleagues. On the other hand, appreciation includes, besides appreciation from supervisors, customers/clients and colleagues, also appreciation from family and friends. It seems that nonwork-based sources of appreciation such as family and friends might be more important in order to reduce the negative consequences of job insecurity on irritation, but this effect disappears when psychologists are experiencing conflicts in the working context. Future research could investigate the interaction between different sources of appreciation and social stressors in order to understand whether a distinction between work-based vs. nonwork-based appreciation or between work-based sources of appreciation within the organization (colleagues, supervisors) vs. work-based sources of appreciation outside the organization (customers, clients) can better explain the effects of job insecurity.

### Limitations

In our study, we measured job insecurity using one item only, as has been successfully done in former investigations (e.g., De Witte, 1999; Otto & Dalbert, 2013). However, Sverke et al. (2002) found that multiple-item scales show stronger relationships with outcomes than single-item scales, which might explain why, on a bivariate level, job insecurity was not significantly related to the outcomes (see Table 1). It is also important to note that some of our results were marginally significant. Pritschet, Powell, and Horne (2016) found that in the last decades, psychologists from different subfields have increased the use of this concept, although there are no explicit rules about what exactly is a marginally significant result and what is not. Therefore, some of our results should be interpreted with caution, and more research is needed in order to investigate whether our pattern of results can be replicated in future studies.

Furthermore, the present study was based on a self-report survey. While some authors suggest that self-report surveys could be appropriate depending on the research question (Spector, 2006), we should also consider that results could increase the likelihood of erroneous results due to common method variance (Podsakoff, MacKenzie, Lee, & Podsakoff, 2003). Specifically, when it comes to our main concept of (subjective) job insecurity, our findings could be consolidated by objective data (as provided by companies, for example). It has to be noted, though, that our study aimed at exploring the effects of subjective (i.e. cognitive) job insecurity perceptions, and that first empirical evidence has indicated that subjective unemployment perceptions and objective economic figures are not linked to selected outcomes in the same way (Otto, Mohr, Kottwitz, & Korek, 2016). While some findings have suggested that objective figures (e.g., unemployment rates) are reflected in job insecurity ratings (e.g., Kinnunen & Nätti, 1994; Schwarz, 2012) the findings are inconsistent, and rather indicate that the interplay of objective and subjective parameters seems to be crucial (Otto et al., 2016). This, however, calls for a simultaneous consideration of both data sources.

Another potential limitation is the fact that this study was an online study. This was necessary because after graduation the psychologists left not only “their” university but also their places of residence – some going abroad – making it impossible to invite them to the laboratory to fill in a questionnaire under controlled conditions. Moreover, by only knowing the email addresses of the graduates and not any further personal data, we were able to guarantee them anonymity and confidentiality. Nevertheless, Wright (2005) described in his study that in addition to the positive aspects of online surveys (e.g., access to groups, saving time and costs), problematic effects such as sampling issues may arise. In our case, not all graduates had allowed email addresses to be listed or used for research projects. Another issue described by Wright (2005) is the willingness to answer an online questionnaire. He pointed out that some people are more likely to complete an online survey than others. Such selection effects may become relevant in case that particularly those psychologists who had been in (un)satisfactory working conditions answered our questionnaire, thereby inducing an underestimation of the true relationships. We also do not know under which conditions the questionnaire was answered: It may play a role for our findings if the psychologists filled in the questionnaire after a long working day with difficult social contacts or during vacation. Hence, our results should be taken with some caution and therefore, as suggested by Wright (2005), replications with similar groups are necessary in order to gain a reliable picture.

Another point is that all results we reported in our study were based on a cross-sectional design. This constitutes a limitation to our study, because with our data we cannot make inferences about long-term effects or about causalities.

Finally, we have a relatively small sample, which could reduce the predictive power of our model. Furthermore, our sample is very specific (psychologists only), and we cannot generalize our results to other professional groups. However, the use of this very specific group provides us with more information about the effects of job insecurity and the roles of appreciation and social stressors in a single and social profession. It therefore offers an ideal opportunity for studying the impact of the social context.

### Theoretical and Practical Implications

We contribute to the literature on how job insecurity could affect well-being by pointing at the complex – but often neglected – role of the social context. From a theoretical point of view, we provide knowledge that looking at the social surrounding only from one perspective might indeed lead to wrong predictions. Social professions contain regular interpersonal contacts, and it seems to be essential to consider the whole spectrum of positive and negative experiences.

From the view of practitioners, we do see several key factors to focus on when trying to prevent a drop in well-being. Psychologists in companies, as consultants, or in the academic context are normally professionals who plan interventions, for instance for improving the work climate, in order to increase the well-being of employees. Our study shows that variables of the social environment at the workplace (appreciation and social stressors) have an effect on job satisfaction or dedication to the job of psychologists themselves. Sobiraj et al. (2016) found that appreciation was important for psychologists. Thus, independent of the field where a psychologist is working, it is important to provide appreciation in order to reduce the negative consequences of job insecurity on well-being.

To build an organizational culture that provides appreciation and minimizes conflicts, mobbing or any kind of social undermining (as reflected in social stressors) is an important goal to sustain well-being. Since conflicts cannot always be avoided, it would be important to learn strategies for how to better cope with such kinds of social stressors at work, how to deal with conflicts, and how to provide constructive feedback. Hence, both kinds of interventions, i.e., structural- and behaviour-oriented interventions should be combined. In behaviour-oriented trainings, psychologists or other people in social professions could learn how to balance out the positive and negative sides of social contacts. When it comes to structural interventions, a positive social work environment should be established that should also be open to provide realistic information about the security of one’s job.

### Conclusions

To sum up, this study extends the knowledge about the negative consequences of job insecurity considering the social context – in particular exploring its role in people working in a social profession. As the social context can provide both resources and stressors, we explored how job insecurity affects three indicators of well-being: satisfaction, irritation, and dedication, considering their dependence on the complex interplay of appreciation for one’s work and conflicts (social stressors). In doing so, we provide more information on variables of the social framework which could shape the effects of job insecurity on professionals’ well-being.
